# Docosahexaenoic fatty acid reduces the pro‐inflammatory response induced by IL-1β in astrocytes through inhibition of NF-κB and AP-1 transcription factor activation

**DOI:** 10.1186/s12868-021-00611-w

**Published:** 2021-01-27

**Authors:** Emilia Zgórzyńska, Dawid Stulczewski, Barbara Dziedzic, Kuan-Pin Su, Anna Walczewska

**Affiliations:** 1 Department of Cell-to-Cell Communication, Medical University of Lodz, Mazowiecka 6/8, 92- 215 Lodz, Poland; 2 An-Nan Hospital, China Medical University, Tainan, Taiwan; 3 College of Medicine, China Medical University, Taichung, Taiwan; 4 Department of Psychiatry and Mind-Body Interface Laboratory, China Medical University, Taichung, Taiwan

**Keywords:** Neuroinflammation, Astrocytes, Docosahexaenoic acid

## Abstract

**Background:**

Astrocytes are responsible for a broad range of functions that maintain homeostasis in the brain. However, their response to the pro-inflammatory cytokines released by activated microglia in various neurological pathologies may exacerbate neurodegenerative processes. Accumulating evidence suggests that omega-3 docosahexaenoic fatty acid (DHA) has an anti-inflammatory effect in various cell cultures studies and in a variety of neurological disorders. In this study we examined the mechanism involved in the inhibition of the pro-inflammatory response by DHA in astrocytes treated with IL-1β.

**Methods and results:**

Activation of the transcription factors NF-κB and AP-1 was measured in IL-1β-treated primary astrocytes incubated with various concentrations of DHA. COX-2 and iNOS protein expression was determined by Western blot, and TNF-α and IL-6 secretion was measured using ELISA-based assays. DHA treatment inhibited translocation of p65NF-κB to the nucleus, significantly lowered p65NF-κB protein level and fluorescence of p65NF-κB in the nucleus, reduced dose-dependently IκB protein phosphorylation, and the binding of the AP-1 transcription factor members (c-Jun/c-Fos) to the specific TPA-response element (TRE) of DNA. In addition, the expression of pro-inflammatory COX-2 and iNOS proteins was downregulated and TNF-α and IL-6 secretion was also reduced.

**Conclusions:**

These results indicate that DHA is a powerful factor that reduces the pro-inflammatory response in astrocytes. Consequently, successful introduction of DHA into the astrocyte membranes can attenuate neuroinflammation, which is a key factor of age-related neurodegenerative disorders.

## Background

Neuroinflammation, as a consequence of intensified microglia activation, can trigger, or exacerbate some neurodegenerative disorders [1, 2]. Acute neuroinflammation occurs upon brain trauma, ischemic stroke or infection [3]. Less intense, but longer-lasting chronic activation of glial cells arised from oxidative stress occurring during diminished mitochondrial biogenesis, or decreased efficacy of the antioxidant defense system, can also activate an innate immune response in the nervous system, leading to a variety of neurological disorders [4]. Activation of the innate immune cells in the brain results in the transformation of resting astrocytes into reactive astrocytes characterized by chronic excessive production of cytokines and chemokines [5]. This later phenotype of astrocytes can be induced directly by the bacterial cell-wall endotoxin LPS, through the activation of Toll-like receptors TLR-3 and TLR-4, or by microglial cytokines. Reactive astrocytes display upregulation of the complement cascade genes [6] and an enhanced release of pro-inflammatory cytokines, such as IL-1β and TNF-α [7], which are destructive to synapses [8] and can be a feature of chronic pain pathologies [9].

In astrocytes, as in other cell types, the transcription factor NF-κB is a critical regulator of the inflammatory response. In the canonical activation of the NF-κB heterodimer, the IκB inhibitor is phosphorylated by IκB kinase (IKK) following activation by tumor necrosis factor receptor (TNF-R)-associated factors (TRAFs) that bind to the cytoplasmic regions of variety receptors, e.g. the IL-1/TLR superfamily of receptors [10].

We have previously reported that the incorporation of docosahexaenoic fatty acid (DHA) into astrocyte plasma membranes results in the translocation of nuclear factor erythroid-2-related factor 2 (Nrf2) to the nucleus and markedly reduces the level of spontaneous and challenged reactive oxygen species (ROS) [11]. Since oxidative stress is a powerful factor of NF-κB activation [12] and cross-talk has been reported between the Nrf2 and NF-κB pathways [13, 14], in the present study we examined whether DHA can inhibit NF-κB activation and a pro-inflammatory response in astrocytes.

Our previous findings indicate that p38 mitogen-activated protein kinase (p38 MAPK) is involved in the Nrf2-dependent anti-oxidative enzyme expression in DHA-enriched astrocytes [11], so in the present study we examined activation of AP-1 transcription factor members that can be phosphorylated by MAP kinases. The AP-1 dimeric complex is required for the transactivation of genes encoding both Nrf2-dependent anti-oxidative enzymes and NF-κB-dependent pro-inflammatory mediators [15, 16]. Our findings confirm that presence of increased DHA content in membrane phospholipids downregulates the cyclooxygenase-2 (COX-2) and inducible nitric oxide synthase (iNOS) protein expression and attenuates the release of pro-inflammatory cytokines from astrocytes via suppression of AP-1 and NF-κB activation.

## Methods

### Isolation and culture of primary astrocytes

Primary astrocytes were isolated from the cortex of 1–2 day-old rat pups as described previously [11]. The protocol was approved by the local ethics committee. Briefly, after decapitation without anesthesia to not chemically contaminate tissues, the brain was dissected, washed with PBS and the meninges were stripped off. The cortices were dissociated by trituration in sterile tubes with DMEM. The suspension was filtered forcefully through 180 µm and 30 µm Nitex mesh (Merck, Millipore), and centrifuged at RT. The cell pellet was resuspended in DMEM:F12 medium containing 10 % FBS and cells were seeded at a density 2 × 10^5^ cells/cm^2^ into 75 cm^2^ culture flasks. The medium was changed every two days up to a confluence of 70–80 % and then the flasks were shaken to detach the non-astrocytic cells. Residual cells were digested with 0.25 % trypsin, counted, plated into 75 cm^2^ flasks and cultured until they were 80 % confluent. The purity of the astrocyte culture was 99 %, as determined by GFAP fluorescent immunostaining.

### Western blotting analysis

The expression of p65 NF-κB and pro-oxidative enzymes was quantified in astrocytes incubated with DHA and treated with 1L-1β (10 ng/ml) for one hour and 24 h, respectively. Next, the cells were washed with PBS and lysed in M-PER Reagent for whole-cell lysate and in NE-PER Reagents (Thermo Fisher Scientific) for cytoplasmic and nuclear fractions. The protein samples (30 µg) were separated on SDS–PAGE, transferred onto nitrocellulose membranes (Millipore) and assayed with anti-p65 NF-κB, anti-COX-2 and anti-iNOS antibodies (Santa Cruz Biotechnology, Inc.). Immune complexes were detected with a Super Signal West chemiluminescent reagent (Thermo Scientific–Pierce). Anti-β-actin and anti-histone H3 (Santa Cruz Biotechnology, Inc.) antibodies were used as a loading control. The immune-reactive signals were quantified by densitometry using Image-J software and normalized to β-actin, or histone H3. Western blot analysis was performed in duplicate. The total protein concentration was measured by modified Lowry’s method and the concentration was read from a BSA standard curve.

### Immunofluorescence

Cells cultured on glass coverslips were treated in the same way as for p65 NF-κB Western Blot analysis, then washed with PBS and fixed with 4 % paraformaldehyde in PBS at RT. Next, they were permeabilized with 0.2 % Triton X-100, blocked with 2 % serum in PBS and incubated overnight at 4 °C with anti-p65 NF-κB (1:200). After washing with PBS, and Alexa 594-conjugated secondary antibodies (Thermo Fisher Scientific) were applied at RT, in the dark, for two hours. Finally, the cells were co-stained with the DNA-binding dye 4,6-diamidino-2-phenylindole (DAPI), and the coverslips were mounted on glass microscope slides using ProLong Gold antifade reagent. Fluorescent images were acquired with an AxioExaminer epifluorescence microscope (Carl Zeiss, Oberkochen, Germany) equipped with a water immersion objective. Images were captured at 40x magnification.

### Enzyme‐linked immunosorbent assay (ELISA) measurement of phosphorylated IκBα

Astrocytes were plated into 100 mm Petri dishes at a density of 1.1 × 10^6^ and incubated with 10, 30, 50 µM DHA for 24 h, followed by one-hour treatment with IL-1β (10 ng/ml). The level of phosphorylated IκBα (pIκBα) in whole-cell lysates was quantified using the kit IκBα (pS32/36) + Total IκBα Simple Step ELISA Kit (Abcam) according to the manufacturer’s protocols. Briefly, an antibody cocktail was added to the samples in strip wells, shaken (400 rpm) at RT for one hour. Following this, the wells were washed and samples were incubated with TMB Substrate (3,3′,5,5′-Tetramethylbenzidine) on a shaker in the dark, for 15 min. Reaction catalyzed by HRP was stopped and the intensity of the colorimetric signal was recorded at 450 nm in a Victor^2^ microplate reader (Perkin-Elmer). The measurements were carried out in triplicate.

### Quantifying of AP-1 binding to DNA

The astrocytes cultured in 150 mm Petri dishes (2.4 × 10^6^) were incubated with various concentrations of DHA and treated for 4 hours with IL-1β. Nuclear proteins were extracted using NE-PER Reagent (Thermo Fisher Scientific) according to the manufacturer’s instruction. Binding of c-Fos and c-Jun to DNA was determined with a colorimetric kit (Active Motif). Briefly, the nuclear extracts were added to a 96-well plate pre-coated with oligonucleotides containing the consensus binding sites for AP-1, then antibodies against c-Fos and c-Jun were added and the plate was incubated at RT for 1 hour. After washing and incubation with HRP-conjugated secondary antibodies for 1 hour, the developing solution was added and the reaction was terminated by Stop Solution. The absorbance at OD 450 nm was read within 5 minutes.

### TNF-α and IL-6 determination

Astrocytes were pre-incubated with various concentrations of DHA for 24 hours and challenged with IL-1β (10 ng/ml) for four hours. The culture media was taken and then assayed for IL-6 and TNF-α levels using commercially-available Quantikine ELISA Kits (R&D Systems).

### Statistical analysis

The results are given as means ± SEM. All data were analysed using GraphPad Prism 6.0 software. Statistical significance was determined by one-way ANOVA with Bonferroni’s correction. For non-parametric data, the Kruskal–Wallis test was applied, followed by Dunn’s multiple comparison test. The level of significance was set at p < 0.05.

## Results

### NF-κB translocation into the nucleus

**Fig. 1:**
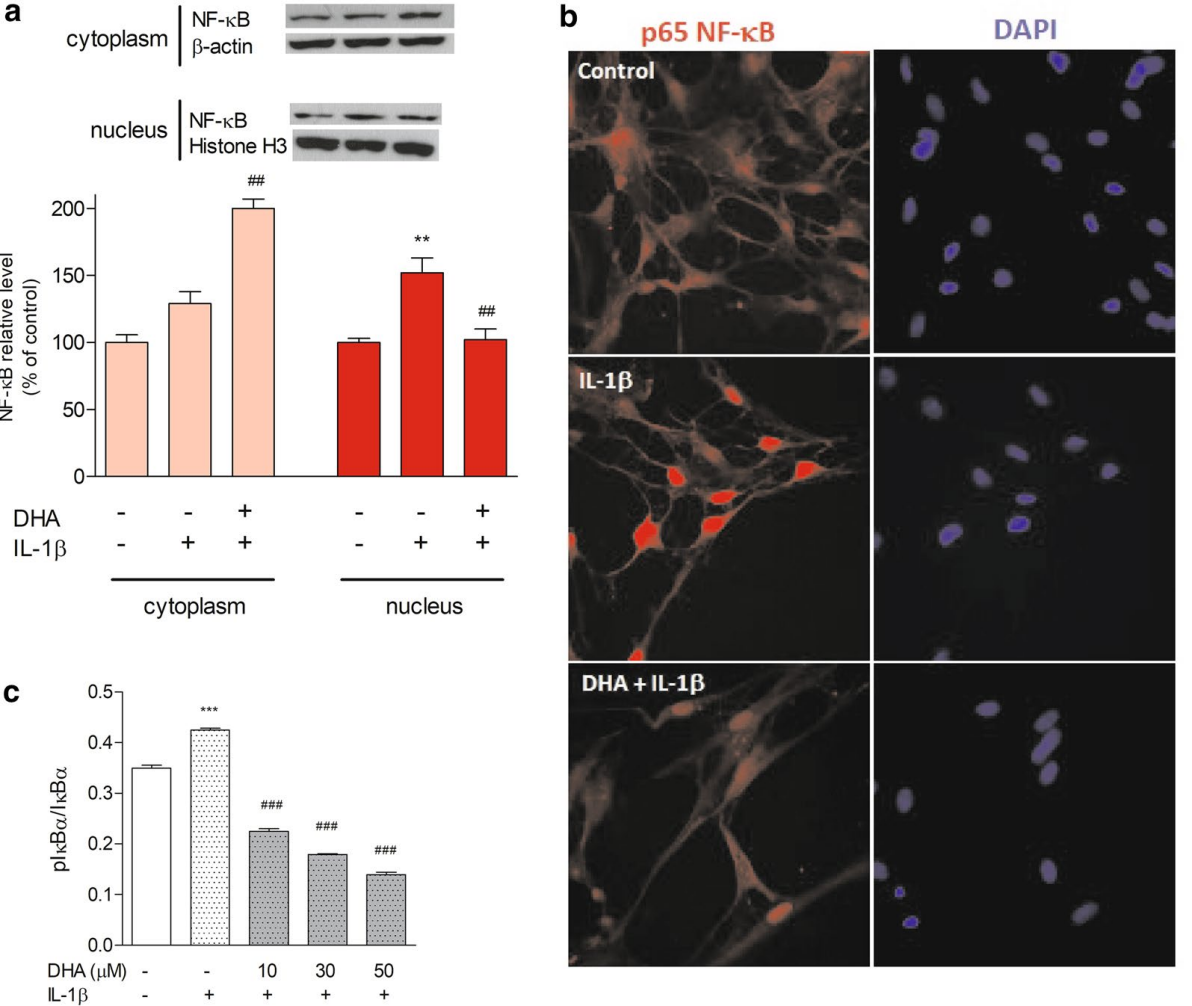
The NF-κB and phosphorylated IκBα (pIκBα) levels in DHA-enriched astrocytes treated with IL-1β. **a** The level of NF-κB was assessed by Western blot in the cytoplasmic and nuclear fractions of astrocytes incubated with, or without, 30 µM DHA for 24 h, followed by treatment with IL-1β (10 ng/ml) for 1 h. Cells untreated with DHA and IL-1β were used as controls. Full-length blots are presented in Additional file 1: Fig. S1. **b** NF-κB (red) and DAPI (blue) immunofluorescence in astrocytes treated as described above. **c** Dose-dependent inhibition of IκBα phosphorylation by DHA. The cells were incubated with various concentrations of DHA and treated with IL-1β. The data are given as means ± SEM. **p < 0.01, ***p < 0.001 compared to control, ^##^p < 0.01, ^###^p < 0.001 compared to IL-1β-treated cells

The immunoblotting indicated that the NF-κB level increased by 29 % in the cytoplasmic fraction and 50 % in the nuclear fraction in IL-1β-treated astrocytes compared to control cells (Fig. 1a). However, incubation with DHA inhibited accumulation of NF-κB in the nucleus (p < 0.01) with a concomitant increase in the cytoplasm (p < 0.01). Consistent with the Western blot analysis, the immunofluorescence assay showed that IL-1β-mediated nuclear translocation of NF-κB to the nucleus was considerably reduced after incubation of astrocytes with DHA (Fig. 1b).

Since IκB is the key inhibitor of the IKK/NF-κB signalling pathway, we measured the levels of total and phosphorylated IκB in astrocytes incubated with DHA and then treated with IL-1β. The ratio of pIκB/total IκB was already decreased in astrocytes incubated with the lowest DHA concentration (from 0.42 ± 0.004 to 0.22 ± 0.005). Incubation with higher concentrations of DHA resulted in a greater decrease in phosphorylated kappa B inhibitor level. The ratio of pIκB/total IκB was reduced to 0.18 ± 0.002 and 0.14 ± 0.005 in 30 µM and 50 µM DHA-treated cells, respectively.

The dose-dependent DHA inhibition of pIκB level is shown in Fig. 1c.

### c-Jun and c-Fos binding to DNA sequences

**Fig. 2:**
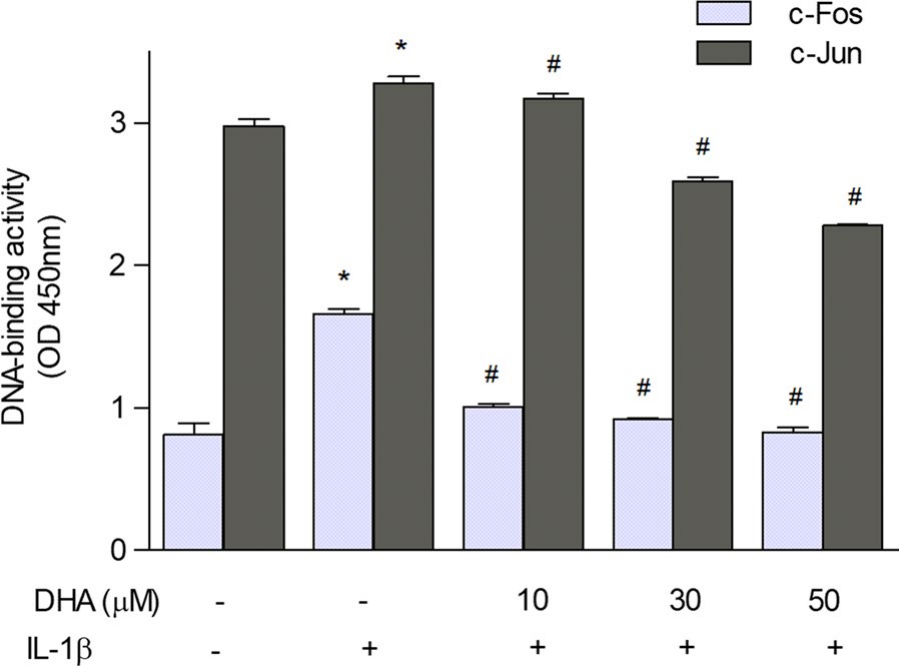
Binding of c-Jun and c-Fos to the TRE motif in DNA, in DHA-enriched astrocytes treated with IL-1β. An ELISA-based (Trans-AM) method was used to quantify the transcription factors binding to DNA in astrocytes incubated with various concentrations of DHA, followed by 4-hour incubation with IL-1β (10 ng/ml). The data are given as means ± SEM from two independent experiments. *p < 0.001 compared to control; ^#^p < 0.001 compared to IL-1β-treated cells

As c-Fos and c-Jun bind predominantly to the TPA-responsive element (TRE) motif in DNA regulating a broad array of genes, oligonucleotides with the TRE sequence were used to determine whether the binding of the AP-1 members to DNA is reduced in DHA-enriched astrocytes. Indeed, the binding was significantly reduced in a dose-dependent manner in cells incubated with DHA and treated with IL-1β compared to those not preincubated with DHA (Fig. 2).

### COX-2 and iNOS protein expression and cytokine release

**Fig. 3:**
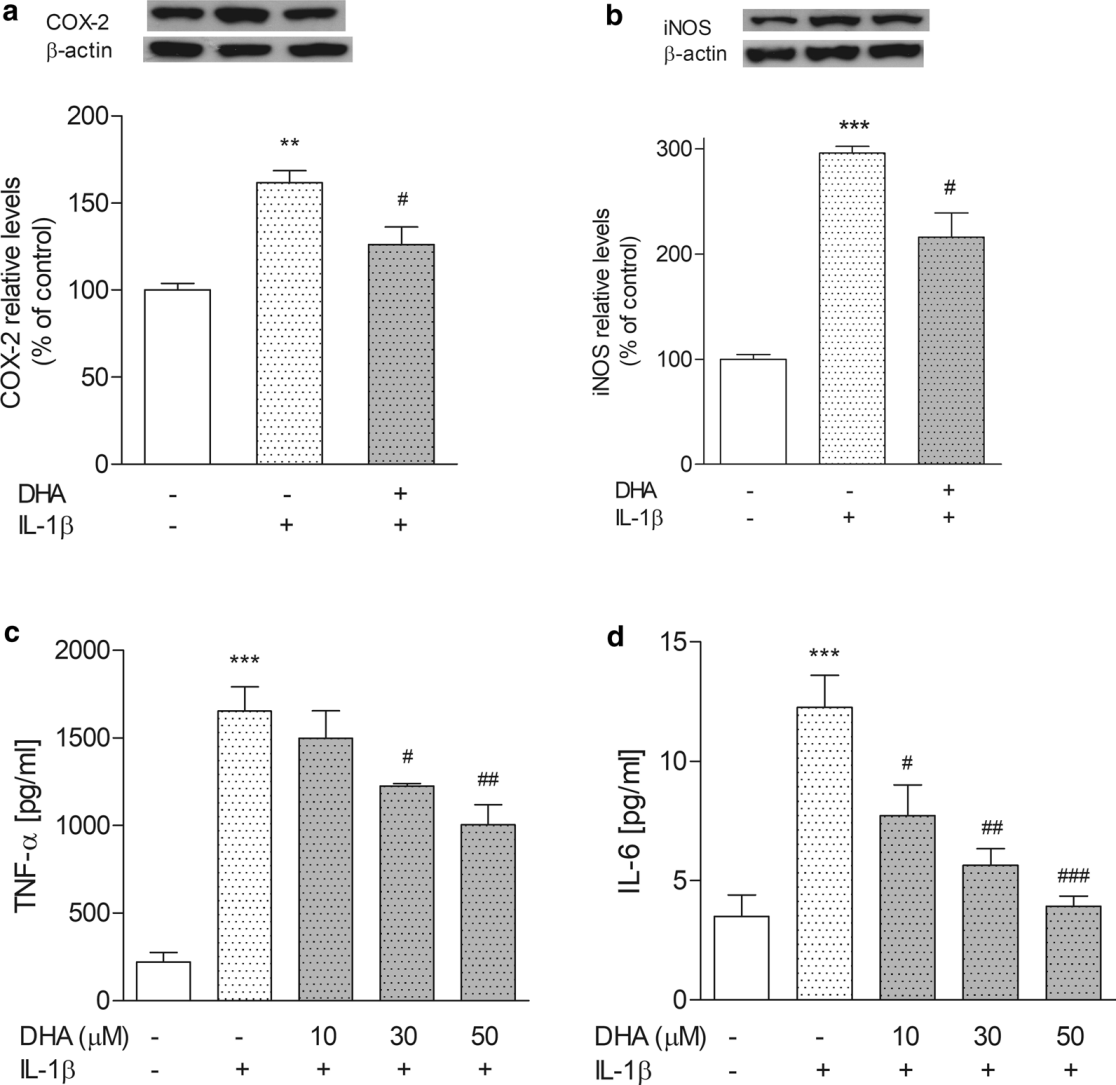
The level of COX-2 and iNOS proteins (**a**, **b**) and TNF-α and IL-6 (C and D) in astrocytes. Proinflammatory enzyme expression was determined in whole cell lysate after incubation of cells with 30 µM DHA, followed by IL-1β (10 ng/ml) treatment. Full-length blots are presented in Additional file 2: Fig. S2. The concentration of cytokines was measured twice in triplicate in media from astrocytes incubated with various concentrations of DHA and treated with IL-1β using an ELISA-based method. The data are given as means ± SEM. **p < 0.01, ***p < 0.001 compared to control, ^#^p < 0.05, ^##^p < 0.01, ^###^p < 0.001 compared to IL-1β-treated cells

Astrocytes incubated with 30 µM DHA followed by IL-1β treatment demonstrated significantly lower COX-2 protein levels, as a percentage of control values (126 ± 10.1 %) compared to cells not incubated with DHA (162 ± 6.8 %) (Fig. 3a). DHA loading also significantly suppressed IL-1β-induced iNOS expression. The iNOS level was 216 ± 22.0 % in DHA compared to the group treated with IL-1β without pretreatment with DHA (296 ± 6.5 %) (Fig. 3b). In addition, the TNF-α level was 1654 ± 138.4 pg/ml in cells treated with IL-1β without DHA pretreatment, while was reduced to 1499 ± 156.2 pg/ml, 1226 ± 12.4 pg/ml and 1004 ± 114.9 pg/ml in cells incubated with 10, 30 and 50 µM DHA, respectively. Secretion of IL-6 decreased from 12.3 ± 1.35 pg/ml in IL-1β-treated cells without incubation with DHA to 7.7 ± 1.28 pg/ml after incubation with 10 µM DHA and to 3.9 ± 0.43 pg/ml following incubation with 50 µM DHA (Fig. 3d).

## Discussion

It is widely reported that dietary supplementation with fish oil, containing omega-3 polyunsaturated DHA, has immune-modulatory effects [17] and inhibits glial cell activation [18] that ameliorates symptoms of neuroinflammatory disorders in the nervous system [19, 20]. Our previous findings showed a tenfold increase in DHA content in the cell membrane compared to unexposed control cells and Nrf2-dependent upregulation of antioxidant enzyme expression associated with decrease in the intracellular ROS level [11]. The present results show that in DHA-incubated astrocytes NF-κB activation was markedly diminished after IL-1β treatment, with a concomitant lowering of IκB phosphorylation, indicating decreased IKK activity. Phosphorylation of is essential for the polyubiquitination of IκB and 26S proteasomal degradation resulting in NF-κB translocation to the nucleus, where it binds to the IκB motifs in the target genes, mostly involved in inflammatory response. DHA treatment downregulated pro-inflammatory enzyme expression and reduced the release of TNF-α and IL-6, indicating that DHA-enrichment limited the pro-inflammatory response to IL-1β treatment in astrocytes. In addition, attenuation of excessive release of pro-inflammatory cytokines by DHA could also reduce the pain sensation associated with ongoing neuroinflammation. Some evidence indicates that pro-inflammatory cytokines drive neuropathic pain [21] so decreased release of inflammatory mediators by activated astrocytes can also be helpful in reducing neuroinflammation-related pain.

The inhibition of the IL-1β-induced pro-inflammatory response by DHA can either occur directly due to modification of the membrane composition, resulting in the modulation of signaling pathways, or indirectly through fatty acid derivatives. The administration of DHA as a dietary supplement [22, 23], or into cells in culture [24] decreases arachidonic acid levels and increases DHA content in membrane phospholipids. As a result of membrane modification, in response to lipoxygenase activation diminish the production of pro-inflammatory arachidonic acid derivatives and increase DHA derivatives levels, such as neuroprotectin D1 and maresin 1, which ameliorate neuroinflammation [25, 26]. Since that the pro-inflammatory enzymes were found to be downregulated in DHA-enriched astrocytes, we hypothesize that the anti-inflammatory action of DHA is most likely associated with a reorganization of lipid microdomains modifying the activity of signaling proteins associated with the cell membrane.

Most mammalian cells, besides the neurons and retina, have negligible levels of DHA in their plasma membranes [27]. However, the incubation of different cell types with fish oil, or preformed omega-3 fatty acid increases the DHA content in the membranes [28]. A high proportion of DHA-containing phospholipids modifies lipid-protein interaction and the signaling pathways from the cell membranes to target cellular effectors. Following activation by ligands, the IL-1β receptors bind the MyD88 adaptor, which forms a complex with IRAKs and TRAF6. In the complex, TRAF6 possessing E3 ubiquitin ligase activity polyubiquitinates IRAK1 and the TRAF6/IRAK complex dissociates from MyD88. The polyubiquitinated IRAK undergoes proteasomal degradation and TRAF6 activates TAK1 (MAP3K) to form a functional complex with TAB1 and TAB2 proteins (TAK binding proteins) [29]. TAK1 acts as a relay for MKKs/AP-1 and NF-κB transcription factor activation. Thus, the attenuation of TAK1 activity in DHA-enriched astrocytes by upstream signaling molecules is most probably involved in a suppression of NF-κB and AP-1 transcription factor activation. Such mechanism is in agreement with previous findings that both omega-3 DHA and EPA suppressed LPS-induced ubiquitination and dislocation of TRAF6 from the myddosome to the cytoplasm, and inhibited the phosphorylation of TAK1 and its downstream effectors, p38 MAPK and IκBα proteins [30].

Various mechanisms can be involved in the inhibition of TAK1 associated with increases in DHA phospholipids in membranes. The incorporation of DHA into phospholipid species, mostly to phosphatidylethanolamine and phosphatidylserine (PS), has been found to re-organize lipid rafts, thus modifying downstream signaling pathways [31, 32]. As a result, anionic PS molecules provide docking sites for a range of proteins, such as the small GTPases from the Ras family that modify downstream signaling via the Raf-1/MAPKs kinase cascades [33]. The incorporation of DHA acyls into phospholipids may modify the activity of the membrane associated Raf kinase inhibitor (RKIP), which constitutively inhibits the Raf-1 and downstream MEK/ERK pathway [34, 35]. In addition, RKIP can interact with TAK1, antagonizing the activation of IKK [36]. The function of RKIP can be modified via phosphorylation through kinases such as PKC that transform RKIP hindering its interaction with downstream kinases. As DHA is known to inhibit the catalytic subunit of PKC [37], and PKC contributes to the inhibition of both the Raf-1/MEK/ERK and TAK1/IKK/NF-κB pathways, so this may be a mechanism of inhibition of NF-κB and AP-1 activity. Alternatively, the DHA-activated G-protein coupled receptor 120 (GPR120) can form a complex with β-arrestin 2, which after internalization into the cytoplasm can bind to TAB1 and inhibits TAK1/NF-κB activation [38].

## Conclusions

The present findings indicate that DHA-enriched astrocytes display a considerably reduced response to IL-1β resulted from an inhibition of NF-κB and AP-1 transcription factor activation, a diminution of iNOS and COX-2 levels and reduction of pro-inflammatory TNF-a and IL-6 release. IL-1 receptor activation contributes to transformation of astrocytes to their reactive phenotype which creates milieu for neurological disorders and neurodegenerative diseases, such as Parkinson’s and Alzheimer`s disease. Therefore, we hypothesize that omega-3 DHA may limit neuropathological processes by suppressing astrocyte reactivity to inflammatory insults.

## Supplementary Information

**Additional file 1: Fig. S1.** Unprocessed images of Western blots (A and B design two independent experiments) for NF-κB in the cytoplasmic and nuclear fractions of astrocytes incubated with 30 µM DHA for 24 hours, followed by treatment with IL-1β (10 ng/ml) for 1 h.

**Additional file 2: Fig. S2.** Unprocessed images of Western blots of COX-2 and iNOS in whole cell lysates after incubation of astrocytes with 30 µM DHA, followed by activation with IL-1β (10 ng/ml).

## Data Availability

All data are included in this article.

## Abbreviations

AP-1: activator protein 1
ARE: antioxidant response element
c-Fos, c-Jun: subunits of AP-1transcription factor
COX-2: cyclooxygenase-2
DHA: docosahexaenoic acid
ERK: extracellular signal regulated kinase
GPR120: G-protein coupled receptor 120
IκB: inhibitor of NF-κB
IKK: IκB kinase
IL-1: interleukin 1
IL-6: interleukin 6
iNOS: inducible nitric oxide synthase
IRAK: Interleukin-1 receptor associated kinase
MAPK: mitogen-activated protein kinase
MKK: mitogen-activated protein kinase kinase
MyD88: myeloid differentiation factor 88
Nrf2: nuclear factor erythroid*-*2*-*related factor 2
NF-κB: nuclear factor- kappa B
PKC: protein kinase C
Raf-1: serine/threonine kinase 1
Ras: Ras GTPase
ROS: reactive oxygen species
RKIP: Raf kinase inhibitor protein
TAB: TAK- binding protein
TAK1: transforming growth factor-β-activated kinase 1
TNF-α: tumor necrosis factorα
TLRs: Toll-like receptors
TRAFs: tumor necrosis factor receptor associated factors
TRE: TPA-response element
