# Supplementary figures and images for: Docosahexaenoic fatty acid reduces the pro‐inflammatory response induced by IL-1β in astrocytes through inhibition of NF-κB and AP-1 transcription factor activation
Source: BMC Neurosci. 2021 Jan 27;22:4. doi: 10.1186/s12868-021-00611-w (PMC7839194; doi:10.1186/s12868-021-00611-w)

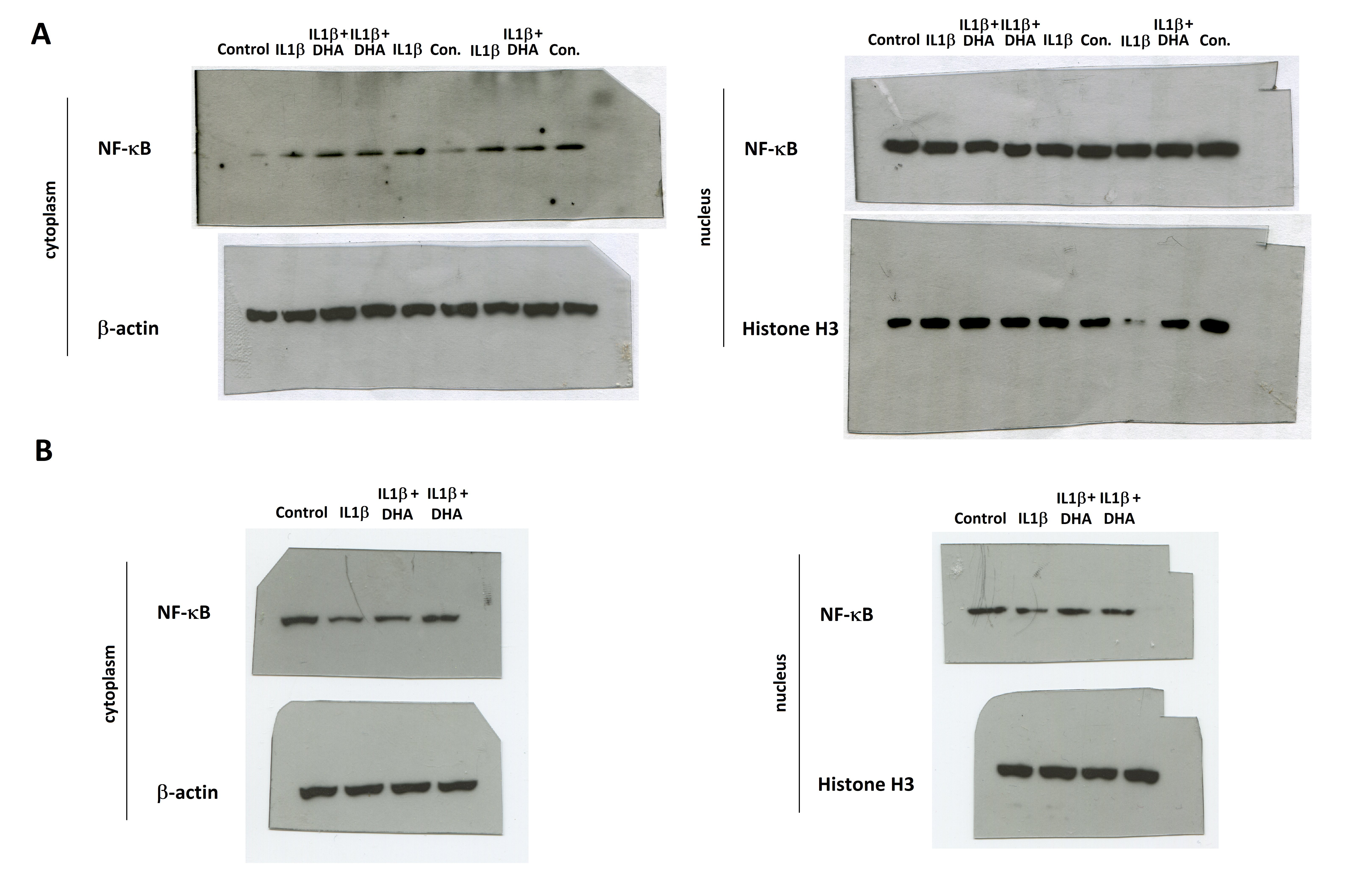

Supplement: Supplementary file 1 — Additional file 1: Fig. S1. Unprocessed images of Western blots (A and B design two independent experiments) for NF-κB in the cytoplasmic and nuclear fractions of astrocytes incubated with 30 µM DHA for 24 hours, followed by treatment with IL-1β (10 ng/ml) for 1 h. [file 12868_2021_611_MOESM1_ESM.tif]

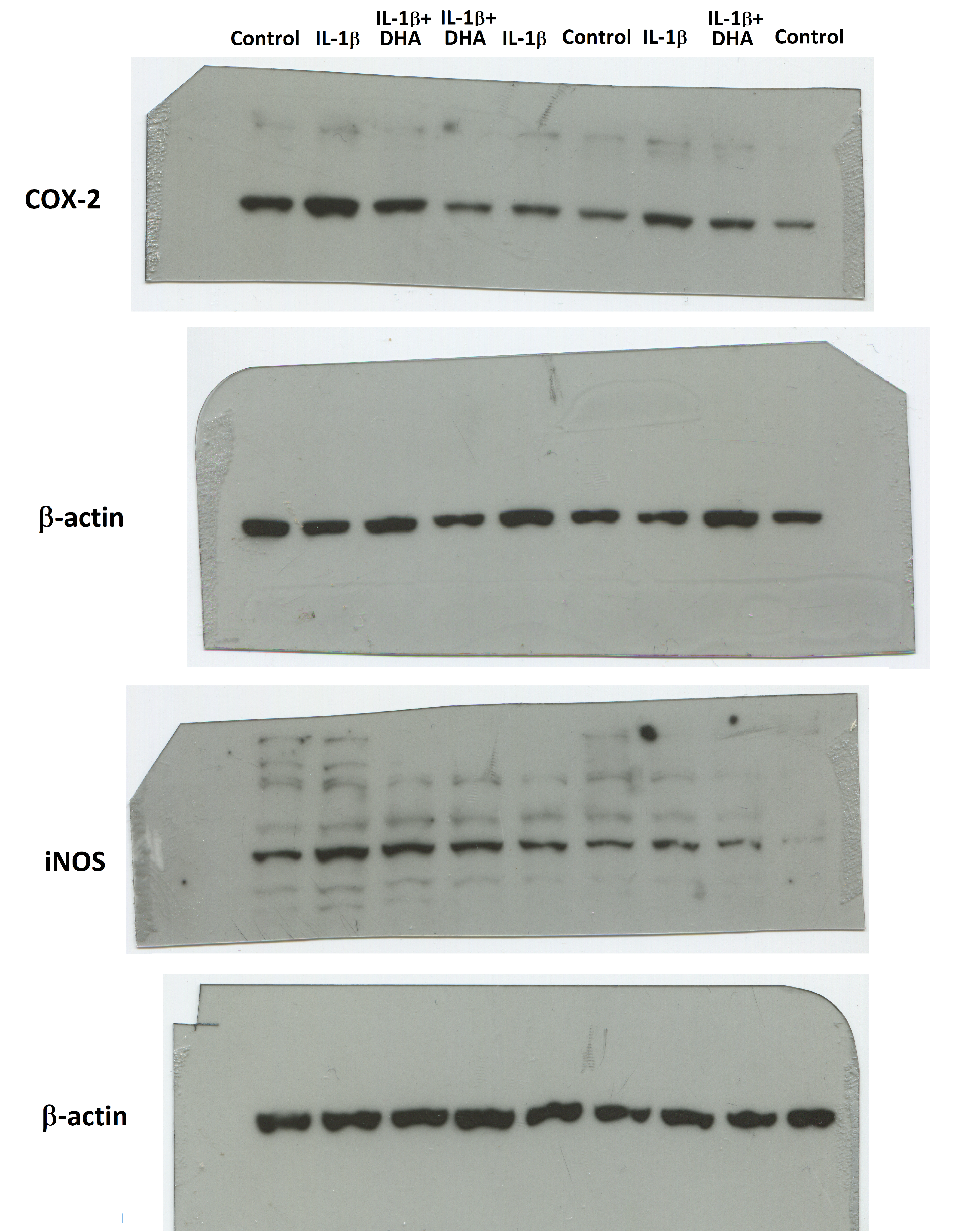

Supplement: Supplementary file 2 — Additional file 2: Fig. S2. Unprocessed images of Western blots of COX-2 and iNOS in whole cell lysates after incubation of astrocytes with 30 µM DHA, followed by activation with IL-1β (10 ng/ml). [file 12868_2021_611_MOESM2_ESM.tif]
